# The Photovoltaic Heat Island Effect: Larger solar power plants increase local temperatures

**DOI:** 10.1038/srep35070

**Published:** 2016-10-13

**Authors:** Greg A. Barron-Gafford, Rebecca L. Minor, Nathan A. Allen, Alex D. Cronin, Adria E. Brooks, Mitchell A. Pavao-Zuckerman

**Affiliations:** 1 School of Geography & Development, University of Arizona, Tucson, AZ, USA; 2 Office of Research & Development; College of Science, Biosphere 2, University of Arizona, Tucson, AZ, USA; 3 Nevada Center of Excellence, Desert Research Institute, Las Vegas, NV, USA; 4 Department of Physics, University of Arizona, Tucson, AZ, USA; 5 Department of Electrical and Computer Engineering, University of Wisconsin-Madison, Madison, WI, USA; 6 Department of Environmental Science & Technology, University of Maryland, College Park, MD, USA

## Abstract

While photovoltaic (PV) renewable energy production has surged, concerns remain about whether or not PV power plants induce a “heat island” (PVHI) effect, much like the increase in ambient temperatures relative to wildlands generates an Urban Heat Island effect in cities. Transitions to PV plants alter the way that incoming energy is reflected back to the atmosphere or absorbed, stored, and reradiated because PV plants change the albedo, vegetation, and structure of the terrain. Prior work on the PVHI has been mostly theoretical or based upon simulated models. Furthermore, past empirical work has been limited in scope to a single biome. Because there are still large uncertainties surrounding the potential for a PHVI effect, we examined the PVHI empirically with experiments that spanned three biomes. We found temperatures over a PV plant were regularly 3–4 °C warmer than wildlands at night, which is in direct contrast to other studies based on models that suggested that PV systems should decrease ambient temperatures. Deducing the underlying cause and scale of the PVHI effect and identifying mitigation strategies are key in supporting decision-making regarding PV development, particularly in semiarid landscapes, which are among the most likely for large-scale PV installations.

Electricity production from large-scale photovoltaic (PV) installations has increased exponentially in recent decades^1^,^2^,^3^. This proliferation in renewable energy portfolios and PV powerplants demonstrate an increase in the acceptance and cost-effectiveness of this technology^4^,^5^. Corresponding with this upsurge in installation has been an increase in the assessment of the impacts of utility-scale PV^4^,^6^,^7^,^8^, including those on the efficacy of PV to offset energy needs^9^,^10^. A growing concern that remains understudied is whether or not PV installations cause a “heat island” (PVHI) effect that warms surrounding areas, thereby potentially influencing wildlife habitat, ecosystem function in wildlands, and human health and even home values in residential areas^11^. As with the Urban Heat Island (UHI) effect, large PV power plants induce a landscape change that reduces albedo so that the modified landscape is darker and, therefore, less reflective. Lowering the terrestrial albedo from ~20% in natural deserts^12^ to ~5% over PV panels^13^ alters the energy balance of absorption, storage, and release of short- and longwave radiation^14^,^15^. However, several differences between the UHI and potential PVHI effects confound a simple comparison and produce competing hypotheses about whether or not large-scale PV installations will create a heat island effect. These include: (*i*) PV installations shade a portion of the ground and therefore could reduce heat absorption in surface soils^16^, (*ii*) PV panels are thin and have little heat capacity per unit area but PV modules emit thermal radiation both up and down, and this is particularly significant during the day when PV modules are often 20 °C warmer than ambient temperatures, (*iii*) vegetation is usually removed from PV power plants, reducing the amount of cooling due to transpiration^14^, (*iv*) electric power removes energy from PV power plants, and (*v*) PV panels reflect and absorb upwelling longwave radiation, and thus can prevent the soil from cooling as much as it might under a dark sky at night.

Public concerns over a PVHI effect have, in some cases, led to resistance to large-scale solar development. By some estimates, nearly half of recently proposed energy projects have been delayed or abandoned due to local opposition^11^. Yet, there is a remarkable lack of data as to whether or not the PVHI effect is real or simply an issue associated with perceptions of environmental change caused by the installations that lead to “not in my backyard” (NIMBY) thinking. Some models have suggested that PV systems can actually cause a cooling effect on the local environment, depending on the efficiency and placement of the PV panels^17^,^18^. But these studies are limited in their applicability when evaluating large-scale PV installations because they consider changes in albedo and energy exchange within an urban environment (rather than a natural ecosystem) or in European locations that are not representative of semiarid energy dynamics where large-scale PV installations are concentrated^10^,^19^. Most previous research, then, is based on untested theory and numerical modeling. Therefore, the potential for a PHVI effect must be examined with empirical data obtained through rigorous experimental terms.

The significance of a PVHI effect depends on energy balance. Incoming solar energy typically is either reflected back to the atmosphere or absorbed, stored, and later re-radiated in the form of latent or sensible heat (Fig. 1)^20^,^21^. Within natural ecosystems, vegetation reduces heat gain and storage in soils by creating surface shading, though the degree of shading varies among plant types^22^. Energy absorbed by vegetation and surface soils can be released as latent heat in the transition of liquid water to water vapor to the atmosphere through evapotranspiration – the combined water loss from soils (evaporation) and vegetation (transpiration). This heat-dissipating latent energy exchange is dramatically reduced in a typical PV installation (Fig. 1 transition from A-to-B), potentially leading to greater heat absorption by soils in PV installations. This increased absorption, in turn, could increase soil temperatures and lead to greater sensible heat efflux from the soil in the form of radiation and convection. Additionally, PV panel surfaces absorb more solar insolation due to a decreased albedo^13^,^23^,^24^. PV panels will re-radiate most of this energy as longwave sensible heat and convert a lesser amount (~20%) of this energy into usable electricity. PV panels also allow some light energy to pass, which, again, in unvegetated soils will lead to greater heat absorption. This increased absorption could lead to greater sensible heat efflux from the soil that may be trapped under the PV panels. A PVHI effect would be the result of a detectable increase in sensible heat flux (atmospheric warming) resulting from an alteration in the balance of incoming and outgoing energy fluxes due to landscape transformation. Developing a full thermal model is challenging^17^,^18^,^25^, and there are large uncertainties surrounding multiple terms including variations in albedo, cloud cover, seasonality in advection, and panel efficiency, which itself is dynamic and impacted by the local environment. These uncertainties are compounded by the lack of empirical data.

We addressed the paucity of direct quantification of a PVHI effect by simultaneously monitoring three sites that represent a natural desert ecosystem, the traditional built environment (parking lot surrounded by commercial buildings), and a PV power plant. We define a PVHI effect as the difference in ambient air temperature between the PV power plant and the desert landscape. Similarly, UHI is defined as the difference in temperature between the built environment and the desert. We reduced confounding effects of variability in local incoming energy, temperature, and precipitation by utilizing sites contained within a 1 km area.

At each site, we monitored air temperature continuously for over one year using aspirated temperature probes 2.5 m above the soil surface. Average annual temperature was 22.7 + 0.5 °C in the PV installation, while the nearby desert ecosystem was only 20.3 + 0.5 °C, indicating a PVHI effect. Temperature differences between areas varied significantly depending on time of day and month of the year (Fig. 2), but the PV installation was always greater than or equal in temperature to other sites. As is the case with the UHI effect in dryland regions, the PVHI effect delayed the cooling of ambient temperatures in the evening, yielding the most significant difference in overnight temperatures across all seasons. Annual average midnight temperatures were 19.3 + 0.6 °C in the PV installation, while the nearby desert ecosystem was only 15.8 + 0.6 °C. This PVHI effect was more significant in terms of actual degrees of warming (+3.5 °C) in warm months (Spring and Summer; Fig. 3, right).

In both PVHI and UHI scenarios, the greater amount of exposed ground surfaces compared to natural systems absorbs a larger proportion of high-energy, shortwave solar radiation during the day. Combined with minimal rates of heat-dissipating transpiration from vegetation, a proportionally higher amount of stored energy is reradiated as longwave radiation during the night in the form of sensible heat (Fig. 1)^15^. Because PV installations introduce shading with a material that, itself, should not store much incoming radiation, one might hypothesize that the effect of a PVHI effect would be lesser than that of a UHI. Here, we found that the difference in evening ambient air temperature was consistently greater between the PV installation and the desert site than between the parking lot (UHI) and the desert site (Fig. 3). The PVHI effect caused ambient temperature to regularly approach or be in excess of 4 °C warmer than the natural desert in the evenings, essentially doubling the temperature increase due to UHI measured here. This more significant warming under the PVHI than the UHI may be due to heat trapping of re-radiated sensible heat flux under PV arrays at night. Daytime differences from the natural ecosystem were similar between the PV installation and urban parking lot areas, with the exception of the Spring and Summer months, when the PVHI effect was significantly greater than UHI in the day. During these warm seasons, average midnight temperatures were 25.5 + 0.5 °C in the PV installation and 23.2 + 0.5 °C in the parking lot, while the nearby desert ecosystem was only 21.4 + 0.5 °C.

The results presented here demonstrate that the PVHI effect is real and can significantly increase temperatures over PV power plant installations relative to nearby wildlands. More detailed measurements of the underlying causes of the PVHI effect, potential mitigation strategies, and the relative influence of PVHI in the context of the intrinsic carbon offsets from the use of this renewable energy are needed. Thus, we raise several new questions and highlight critical unknowns requiring future research.

## What is the physical basis of land transformations that might cause a PVHI?

We hypothesize that the PVHI effect results from the effective transition in how energy moves in and out of a PV installation versus a natural ecosystem. However, measuring the individual components of an energy flux model remains a necessary task. These measurements are difficult and expensive but, nevertheless, are indispensable in identifying the relative influence of multiple potential drivers of the PVHI effect found here. Environmental conditions that determine patterns of ecosystem carbon, energy, and water dynamics are driven by the means through which incoming energy is reflected or absorbed. Because we lack fundamental knowledge of the changes in surface energy fluxes and microclimates of ecosystems undergoing this land use change, we have little ability to predict the implications in terms of carbon or water cycling^4^,^8^.

## What are the physical implications of a PVHI, and how do they vary by region?

The size of an UHI is determined by properties of the city, including total population^26^,^27^,^28^, spatial extent, and the geographic location of that city^29^,^30^,^31^. We should, similarly, consider the spatial scale and geographic position of a PV installation when considering the presence and importance of the PVHI effect. Remote sensing could be coupled with ground-based measurements to determine the lateral and vertical extent of the PVHI effect. We could then determine if the size of the PVHI effect scales with some measure of the power plant (for example, panel density or spatial footprint) and whether or not a PVHI effect reaches surrounding areas like wildlands and neighborhoods. Given that different regions around the globe each have distinct background levels of vegetative ground cover and thermodynamic patterns of latent and sensible heat exchange, it is possible that a transition from a natural wildland to a typical PV power plant will have different outcomes than demonstrated here. The paucity in data on the physical effects of this important and growing land use and land cover change warrants more studies from representative ecosystems.

## What are the human implications of a PVHI, and how might we mitigate these effects?

With the growing popularity of renewable energy production, the boundaries between residential areas and larger-scale PV installations are decreasing. In fact, closer proximity with residential areas is leading to increased calls for zoning and city planning codes for larger PV installations^32^,^33^, and PVHI-based concerns over potential reductions in real estate value or health issues tied to Human Thermal Comfort (HTC)^34^. Mitigation of a PVHI effect through targeted revegetation could have synergistic effects in easing ecosystem degradation associated with development of a utility scale PV site and increasing the collective ecosystem services associated with an area^4^. But what are the best mitigation measures? What tradeoffs exist in terms of various means of revegetating degraded PV installations? Can other albedo modifications be used to moderate the severity of the PVHI?

To fully contextualize these findings in terms of global warming, one needs to consider the relative significance of the (globally averaged) decrease in albedo due to PV power plants and their associated warming from the PVHI against the carbon dioxide emission reductions associated with PV power plants. The data presented here represents the first experimental and empirical examination of the presence of a heat island effect associated with PV power plants. An integrated approach to the physical and social dimensions of the PVHI is key in supporting decision-making regarding PV development.

## Methods

### Site Description

**Figure 4:**
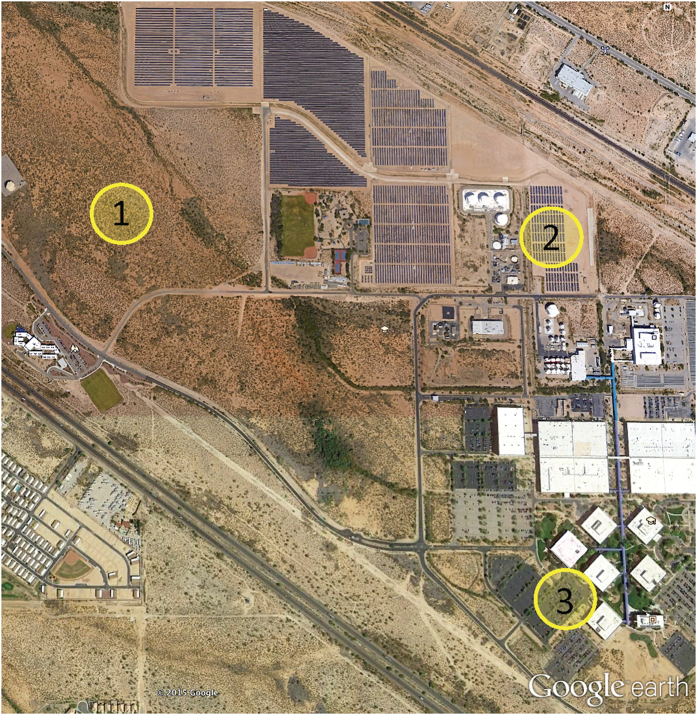
Experimental sites. Monitoring a (1) natural semiarid desert ecosystem, (2) solar (PV) photovoltaic installation, and (3) an “urban” parking lot – the typical source of urban heat islanding – within a 1 km^2^ area enabled relative control for the incoming solar energy, allowing us to quantify variation in the localized temperature of these three environments over a year-long time period. The Google Earth image shows the University of Arizona’s Science and Technology Park’s Solar Zone.

We simultaneously monitored a suite of sites that represent the traditional built urban environment (a parking lot) and the transformation from a natural system (undeveloped desert) to a 1 MW PV power plant (Fig. 4; Map data: Google). To minimize confounding effects of variability in local incoming energy, temperature, and precipitation, we identified sites within a 1 km area. All sites were within the boundaries of the University of Arizona Science and Technology Park Solar Zone (32.092150°N, 110.808764°W; elevation: 888 m ASL). Within a 200 m diameter of the semiarid desert site’s environmental monitoring station, the area is composed of a sparse mix of semiarid grasses (*Sporobolus wrightii, Eragrostis lehmanniana*, and *Muhlenbergia porteri*), cacti (*Opuntia* spp. and *Ferocactus* spp.), and occasional woody shrubs including creosote bush (*Larrea tridentata*), whitethorn acacia (*Acacia constricta*), and velvet mesquite (*Prosopis velutina*). The remaining area is bare soil. These species commonly co-occur on low elevation desert bajadas, creosote bush flats, and semiarid grasslands. The photovoltaic installation was put in place in early 2011, three full years prior when we initiated monitoring at the site. We maintained the measurement installations for one full year to capture seasonal variation due to sun angle and extremes associated with hot and cold periods. Panels rest on a single-axis tracker system that pivot east-to-west throughout the day. A parking lot with associated building served as our “urban” site and is of comparable spatial scale as our PV site.

### Monitoring Equipment & Variables Monitored

Ambient air temperature (°C) was measured with a shaded, aspirated temperature probe 2.5 m above the soil surface (Vaisala HMP60, Vaisala, Helsinki, Finland in the desert and Microdaq U23, Onset, Bourne, MA in the parking lot). Temperature probes were cross-validated for precision (closeness of temperature readings across all probes) at the onset of the experiment. Measurements of temperature were recorded at 30-minute intervals throughout a 24-hour day. Data were recorded on a data-logger (CR1000, Campbell Scientific, Logan, Utah or Microstation, Onset, Bourne, MA). Data from this instrument array is shown for a yearlong period from April 2014 through March 2015. Data from the parking lot was lost for September 2014 because of power supply issues with the datalogger.

### Statistical analysis

Monthly averages of hourly (on-the-hour) data were used to compare across the natural semiarid desert, urban, and PV sites. A Photovoltaic Heat Island (PVHI) effect was calculated as differences in these hourly averages between the PV site and the natural desert site, and estimates of Urban Heat Island (UHI) effect was calculated as differences in hourly averages between the urban parking lot site and the natural desert site. We used midnight and noon values to examine maximum and minimum, respectively, differences in temperatures among the three measurement sites and to test for significance of heat islanding at these times. Comparisons among the sites were made using Tukey’s honestly significant difference (HSD) test^35^. Standard errors to calculate HSD were made using pooled midnight and noon values across seasonal periods of winter (January-March), spring (April-June), summer (July-September), and fall (October-December). Seasonal analyses allowed us to identify variation throughout a yearlong period and relate patterns of PVHI or UHI effects with seasons of high or low average temperature to examine correlations between background environmental parameters and localized heat islanding.

## Additional Information

**How to cite this article**: Barron-Gafford, G. A. *et al*. The Photovoltaic Heat Island Effect: Larger solar power plants increase local temperatures. *Sci. Rep*. **6**, 35070; doi: 10.1038/srep35070 (2016).

## Figures and Tables

**Figure 1:**
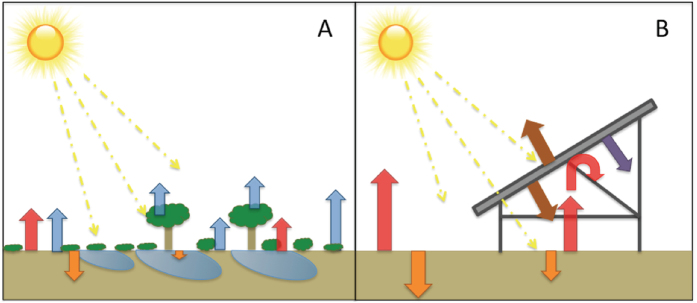
Illustration of midday energy exchange. Assuming equal rates of incoming energy from the sun, a transition from (**A**) a vegetated ecosystem to (**B**) a photovoltaic (PV) power plant installation will significantly alter the energy flux dynamics of the area. Within natural ecosystems, vegetation reduces heat capture and storage in soils (orange arrows), and infiltrated water and vegetation release heat-dissipating latent energy fluxes in the transition of water-to-water vapor to the atmosphere through evapotranspiration (blue arrows). These latent heat fluxes are dramatically reduced in typical PV installations, leading to greater sensible heat fluxes (red arrows). Energy re-radiation from PV panels (brown arrow) and energy transferred to electricity (purple arrow) are also shown.

**Figure 2:**
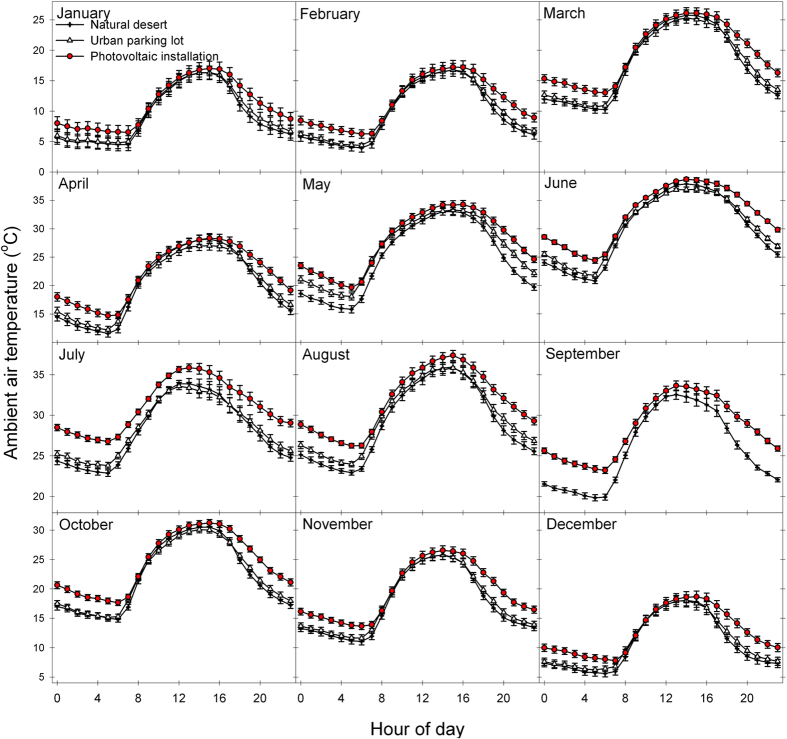
Average monthly ambient temperatures throughout a 24-hour period provide evidence of a photovoltaic heat island (PVHI) effect.

**Figure 3:**
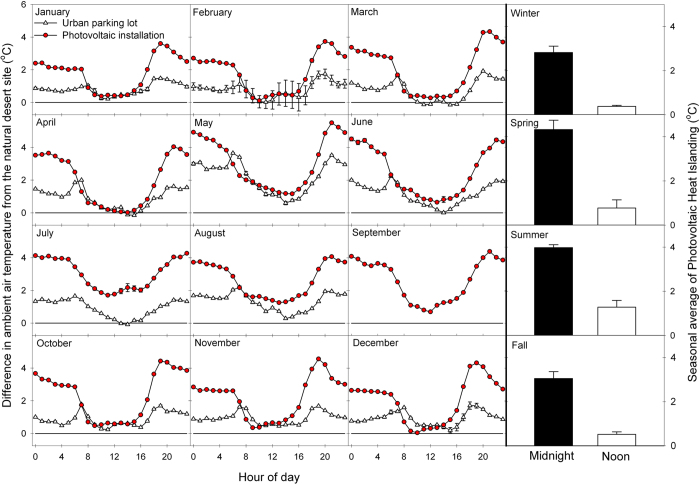
(Left) Average monthly levels of Photovoltaic Heat Islanding (ambient temperature difference between PV installation and desert) and Urban Heat Islanding (ambient temperature difference between the urban parking lot and the desert). (Right) Average night and day temperatures for four seasonal periods, illustrating a significant PVHI effect across all seasons, with the greatest influence on ambient temperatures at night.
